# Association Between Glucocorticoid Exposure and Objective Sleep Architecture in Rheumatoid Arthritis: An Exploratory Pilot EEG Study

**DOI:** 10.3390/jcm15166331

**Published:** 2026-08-16

**Authors:** Shinsuke Yamada, Noriyuki Hayashi, Yuya Fujita, Masao Katsusima, Kazuo Fukumoto, Ryu Watanabe, Motomu Hashimoto

**Affiliations:** 1 Department of Clinical Immunology, Osaka Metropolitan University Graduate School of Medicine, 1-4-3, Asahi-machi, Abeno-ku, Osaka 545-8585, Japan; n24517m@omu.ac.jp (Y.F.); m22647i@omu.ac.jp (M.K.); fkazuoaddress@gmail.com (K.F.); doctorwatanaberyu7@gmail.com (R.W.); motomuhashimoto@gmail.com (M.H.); 2 Department of Internal Medicine, Ohno Memorial Hospital, Osaka 550-0015, Japan; reikoh.impatiens.0725@gmail.com

**Keywords:** rheumatoid arthritis, glucocorticoids, sleep architecture, electroencephalography, delta power, heart rate variability, autonomic dysfunction, digital monitoring

## Abstract

**Objective**: To explore the association of glucocorticoid (GC) exposure with objectively assessed sleep architecture in patients with rheumatoid arthritis (RA). **Methods**: A single-center exploratory pilot study involving 20 consecutive patients with RA (9 GC users and 11 non-users; mean age 64.9 years) was conducted. Using single-channel electroencephalography (EEG) and accelerometry, objective sleep architecture and autonomic balance were evaluated. Sleep parameters included wake after sleep onset (WASO), sleep stages, and delta EEG power during the first sleep cycle, while heart rate variability, expressed as low frequency/high frequency (LF/HF) ratio, was used as an index of autonomic balance. RA disease activity was evaluated using the Disease Activity Score in 28 joints based on C-reactive protein (DAS28-CRP). Associations between clinical variables and objective sleep parameters were evaluated using Spearman rank correlation analysis. **Results**: Disease activity, assessed by DAS28-CRP, did not differ significantly between GC users and non-users. Compared with non-users, GC users had longer WASO (*p* = 0.011), shorter non-rapid eye movement (NREM) stage N3 duration (*p* = 0.010), and lower delta power (*p* = 0.014) than non-users. A higher nighttime-to-daytime LF/HF ratio was also observed in GC users, although this difference did not reach statistical significance. Total sleep time, sleep latency, and sleep efficiency were comparable between the groups. WASO was positively correlated with age, GC use, and GC dose, whereas NREM stage N3 duration and delta power were negatively correlated with GC exposure. No significant associations were observed between age or sex and objective measures of deep sleep. **Conclusions**: Among patients with RA, GC exposure was associated with poorer sleep continuity and reduced deep sleep despite comparable disease activity. Given the small sample size of this exploratory single-center study, these findings should be interpreted cautiously. Larger longitudinal studies using objective sleep measures are warranted to confirm these associations.

## 1. Introduction

Rheumatoid arthritis (RA) is a chronic autoimmune disease characterized by persistent synovial inflammation and progressive joint destruction. Although the introduction of biologic agents and targeted synthetic disease-modifying antirheumatic drugs has markedly improved RA management, glucocorticoids (GCs) remain widely used in routine clinical practice, particularly for patients with an insufficient response or limited access to such advanced therapies [1]. However, despite their potent anti-inflammatory effects, long-term GC exposure is associated with numerous adverse effects, including metabolic [2,3,4], cardiovascular [5,6], and neuropsychiatric [7,8,9] complications.

Among the neuropsychiatric adverse effects of GC therapy, sleep disturbance is among the most common. In the general population, exogenous GC administration has been shown to reduce slow-wave sleep and increase nocturnal awakenings in a dose-dependent manner [10,11,12]. However, most of the available evidence was derived from experimental or non-rheumatologic populations, and the effects of GC exposure on sleep remain largely unexplored in patients with RA.

Approximately 60–80% of patients with RA are affected by sleep disturbances caused by multiple interacting factors, including pain, systemic inflammation, psychological stress, and comorbid conditions [13,14,15]. Importantly, disturbed sleep is also associated with increased pain sensitivity, fatigue, reduced quality of life, and an elevated risk of cardiovascular disease [16,17,18,19]. Nevertheless, despite its clinical importance, objective evaluations of sleep architecture in RA patients remain limited, as most previous studies relied primarily on subjective questionnaires.

Whether GC therapy has an influence on objectively measured sleep structure in patients with RA remains largely unknown. Approximately 20–30% of these patients continue to receive GCs [20] despite guideline recommendations to minimize long-term exposure [21]. Sleep assessment using conventional polysomnography is considered the reference standard, but its complexity and cost limit routine use in clinical practice. In contrast, validated single-channel electroencephalography (EEG) systems provide a practical means for objectively assessing sleep architecture in clinical settings and have shown acceptable agreement with polysomnography. This is the first known objective evaluation of the association between GC exposure and EEG-derived sleep architecture in patients with RA. Accordingly, this exploratory pilot study used single-channel EEG together with heart rate variability analysis in patients with RA to investigate the associations of GC exposure with objective sleep architecture and autonomic balance.

## 2. Methods

### 2.1. Participants

This study included 20 consecutive patients with RA who fulfilled the 2010 American College of Rheumatology/European League Against Rheumatism (ACR/EULAR) classification criteria [22] and were admitted to Osaka Metropolitan University Hospital between November 2016 and February 2018. Patients with clinically diagnosed psychiatric disorders were excluded. Obesity and obstructive sleep apnea syndrome were not exclusion criteria. Body mass index (BMI) and the apnea–hypopnea index (AHI) were assessed in all participants to characterize these potential confounders. At the time of assessment, 9 patients were receiving oral GCs (median dose 7.5 mg/day, interquartile range [IQR] 4.3–10.0) and 3 were using hypnotics (1 GC user, 2 non-users). All GC-treated patients were receiving oral prednisolone for RA. GCs were administered in the morning, with no changes in dose before the sleep assessment. All participants provided written informed consent. The study protocol was approved by the Ethical Committee of Osaka City University Graduate School of Medicine (Approval No. 3506; approved on 30 August 2016) and was conducted in accordance with the Declaration of Helsinki.

### 2.2. Clinical Assessment

Disease activity was assessed using the Disease Activity Score in 28 joints based on C-reactive protein (DAS28-CRP), which is derived from tender joint count, swollen joint count, patient global assessment, and serum C-reactive protein concentration.

### 2.3. Sleep Assessment

Sleep was evaluated at least 2 weeks after hospitalization, once treatment had stabilized and patients had acclimated to the hospital environment. Overnight monitoring was performed using a single-channel EEG device (SLEEP SCOPE; Sleep Well, Osaka, Japan) [23,24,25]. Using disposable electrodes placed over the forehead and mastoid (Fpz–M1 derivation), bipolar EEG signals were recorded at a sampling frequency of 128 Hz. Initially, sleep stages were classified automatically using the validated SLEEP SCOPE algorithm, then reviewed visually by an assessor blinded to the participants’ GC treatment status, according to the manufacturer’s recommended procedure and AASM scoring criteria [26]. The SLEEP SCOPE system has previously been validated against conventional polysomnography for clinical sleep staging [27,28]. Total sleep time (TST) was calculated as sleep duration minus wake after sleep onset (WASO). The first sleep cycle was defined as the interval between sleep onset and the end of the first REM period. Delta power, representing the power spectral density (μV^2^) within the delta frequency band (0.5–2.0 Hz), was used as a marker of deep sleep intensity [29]. This parameter was analyzed as absolute spectral power (μV^2^) without normalization. To account for individual differences in sleep duration and the number of sleep cycles, only delta power during the first sleep cycle was used for analysis [23]. A portable respiratory monitoring system (SAS2100; Nihon Kohden, Tokyo, Japan) was used to assess the AHI by simultaneously recording respiratory airflow, oxygen saturation, and heart rate [23,24,25]. Apnea and hypopnea were defined according to standard criteria, and the AHI was calculated as the number of apnea and hypopnea events per hour of sleep.

### 2.4. Autonomic Function

HRV data were derived from accelerometry (AC-301; GMS Corporation, Tokyo, Japan) [30]. Autonomic nervous system function was evaluated using the low-frequency/high-frequency (LF/HF) ratio derived from HRV, with the nighttime-to-daytime LF/HF ratio used as an index of autonomic balance.

### 2.5. Statistical Analysis

Continuous variables are expressed as median (interquartile range [IQR]), and categorical variables as number (%). Between-group comparisons were performed using the Mann–Whitney U test or Fisher’s exact test, as appropriate. For correlation analyses, GC dose was coded as 0 mg/day in patients who were not receiving GCs. Spearman’s rank correlation analysis was used to evaluate associations between clinical variables and sleep parameters. A two-sided *p* value < 0.05 was considered to be statistically significant. Because this was an exploratory pilot study, no formal sample size calculation was performed. Likewise, no formal adjustment for multiple comparisons was applied, as the primary objective of the study was hypothesis generation rather than confirmatory hypothesis testing. WASO, NREM stage N3 duration, and delta EEG power were considered the principal objective sleep outcomes, with all other analyses regarded as exploratory.

### 2.6. Use of Artificial Intelligence Tools

Generative artificial intelligence (ChatGPT, OpenAI, San Francisco, CA, USA) was used solely to assist with language editing and improve the readability of the manuscript. It was not used for data collection, statistical analysis, interpretation of the results, or generation of scientific conclusions. All AI-assisted content was reviewed and verified by the authors.

## 3. Results

### 3.1. Baseline Characteristics

**Figure 1:**
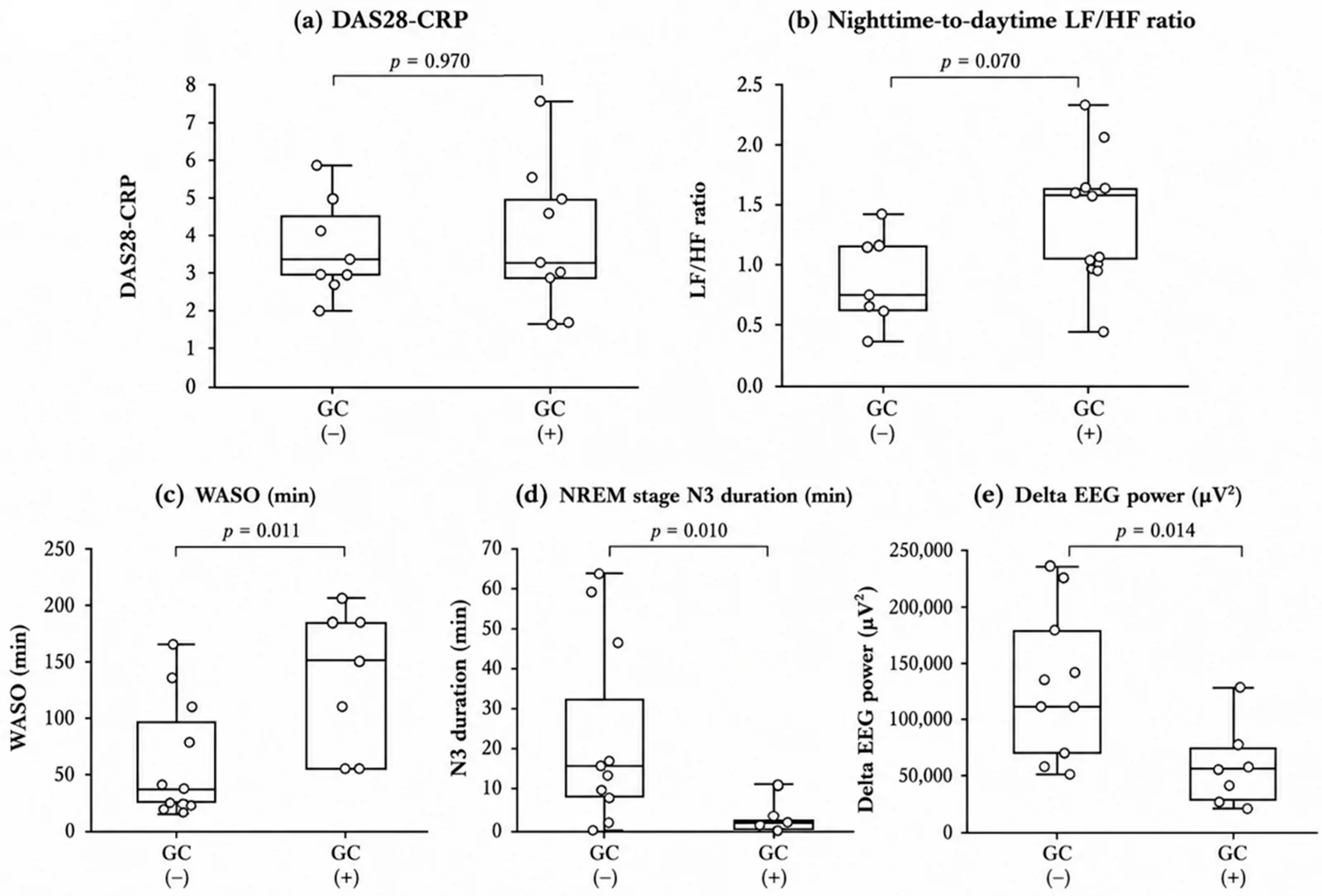
Comparisons of disease activity, autonomic function, and objective sleep parameters between patients receiving glucocorticoids [GC (+)] and those not receiving glucocorticoids [GC (−)]. Individual participant data are shown as jittered circles overlaid on box-and-whisker plots. Boxes represent the interquartile range (IQR), the horizontal line within each box indicates the median, whiskers extend to the most extreme values within 1.5 × IQR, and points beyond the whiskers represent outliers. *p* values were calculated using the Mann–Whitney U test. Abbreviations: DAS28-CRP, Disease Activity Score in 28 joints based on C-reactive protein; LF/HF, low-frequency/high-frequency ratio; WASO, wake after sleep onset; NREM, non-rapid eye movement; EEG, electroencephalography.

The baseline characteristics of GC users and non-users were generally comparable. Among the 20 patients with RA, the median CRP level and DAS28-CRP score at the time of sleep assessment were 0.27 mg/dL and 3.0, respectively. Neither CRP levels nor DAS28-CRP scores differed significantly between the groups (Table 1, Figure 1a).

### 3.2. Objective Sleep Architecture

As compared with non-users, GC users had longer WASO, shorter NREM stage N3 duration, and lower delta power, an objective marker of deep sleep intensity. However, TST, sleep latency, and sleep efficiency did not differ significantly between the groups (Table 1, Figure 1c–e).

### 3.3. Autonomic Balance

GC users tended to have a higher nighttime-to-daytime LF/HF ratio than non-users; however, the difference did not reach statistical significance (Table 1, Figure 1b).

### 3.4. Correlation Analysis Between Sleep Parameters and Clinical Variables

Spearman’s rank correlation analysis identified significant associations between GC exposure and objective sleep parameters. WASO was positively correlated with age, GC use, and daily GC dose, whereas NREM stage N3 duration and delta power were both negatively correlated with GC use and GC dose. BMI showed a modest positive correlation with NREM stage N3 duration, whereas no significant associations were observed with either WASO or delta EEG power. Neither NREM stage N3 duration nor delta power was significantly associated with age, sex, or AHI (Table 2).

### 3.5. Sensitivity Analysis After Excluding Patients Receiving Hypnotics

After excluding the three patients receiving hypnotics, GC users continued to show significantly longer WASO, shorter NREM stage N3 duration, and lower delta EEG power than non-users (Supplementary Table S1).

### 3.6. Exploratory Multivariable Sensitivity Analyses

Separate multivariable linear regression models were constructed to evaluate whether the associations of GC use with objective sleep parameters persisted after adjustment for clinically relevant potential confounders. Because of the limited sample size, age, RA duration, biologic disease-modifying antirheumatic drug (DMARD) use, and AHI were entered individually rather than simultaneously. The association between GC use and lower delta EEG power remained consistent across all exploratory models. For NREM stage N3 duration, the association with GC use was generally preserved, although it was attenuated after adjustment for biologic DMARD use. After adjusting for age or biologic DMARD use, the association between GC use and WASO was attenuated (Supplementary Table S2).

## 4. Discussion

The results of the present objective sleep assessment study suggest that GC exposure may be associated with an impaired sleep architecture characterized by prolonged WASO, reduced NREM stage N3 duration, and decreased delta power in patients with RA. To the best of our knowledge, few studies have used EEG to objectively evaluate the association between GC therapy and sleep architecture in patients with RA. Although disease activity was comparable between GC users and non-users, the cross-sectional design and limited ability to adjust for potential confounders preclude conclusions regarding the independence of these associations from disease activity or other potential confounders.

Delta power is used as a marker of deep sleep intensity and may reflect slow-wave sleep depth, though it is not a direct substitute for polysomnographic staging. Therefore, in this study, delta power was used as a complementary physiological indicator of sleep depth rather than as a direct measure of sleep quality. A major strength of this pilot study is the objective assessment of sleep architecture using EEG. Although sleep disturbances are highly prevalent in RA patients, most previous studies relied on subjective assessment. The present findings provide physiological evidence indicating that GC exposure may be associated with impaired sleep continuity and reduced slow-wave sleep, both of which are essential for restorative sleep. Notably, these differences were observed even at relatively low GC doses (median 7.5 mg/day), thus highlighting their potential clinical relevance.

GC dose was significantly associated with several objective sleep parameters, whereas DAS28-CRP showed no significant associations with these parameters in the present cohort. An exploratory analysis restricted to GC-treated patients showed that higher GC dose was significantly associated with lower delta EEG power (Spearman’s ρ = –0.712, *p* = 0.044), whereas associations with NREM stage N3 duration and WASO did not reach statistical significance (Supplementary Table S3). Given that only nine patients were included in this analysis, these findings should be interpreted with caution. Sleep disturbance in RA has been associated with disease activity and pain [31,32]. The present findings raise the possibility that GC exposure may also be associated with sleep impairment in patients with relatively low disease activity, although this possibility requires confirmation in larger, appropriately adjusted longitudinal studies.

While the present study cannot establish causality, several biological mechanisms may explain the observed association between GC exposure and sleep changes. Exogenous GCs may disrupt the hypothalamic–pituitary–adrenal axis and circadian cortisol rhythms, resulting in relatively elevated nocturnal cortisol levels that may impair sleep maintenance and reduce slow-wave sleep [12,33]. Furthermore, GC users tended to have a higher nighttime-to-daytime LF/HF ratio, which may reflect alterations in nocturnal autonomic regulation, although this difference did not reach statistical significance in this small cohort [34]. Although the LF/HF ratio is widely used as an index of autonomic balance, its interpretation remains controversial [35], and therefore these findings should be interpreted with caution.

Multiple factors contribute to sleep disturbance in RA, including inflammation, pain, and comorbidities [36,37]. However, the comparable DAS28-CRP levels noted in GC users and non-users suggest that inflammation alone is unlikely to account for the observed sleep abnormalities. Pain intensity, assessed using a visual analog scale (VAS), did not differ significantly between GC users and non-users or show a significant association with any of the objective sleep parameters examined. Nevertheless, pain is a multidimensional symptom, and a single VAS assessment may not capture all aspects of pain; therefore, residual confounding related to pain, comedications, and disease duration cannot be completely excluded. Despite these limitations, the consistency of the findings across multiple objective sleep parameters supports the overall coherence of the observed associations.

To further explore the robustness of these findings, exploratory multivariable sensitivity analyses using models adjusted separately for age, RA duration, biologic DMARD use, and AHI were performed. The association with lower delta EEG power remained consistent across all models, while that with reduced NREM stage N3 duration was generally preserved but attenuated after adjustment for biologic DMARD use (Supplementary Table S2). However, given the very small sample size, these analyses should be considered exploratory rather than confirmatory.

In this small cohort, age was positively correlated with WASO, but was not significantly associated with NREM stage N3 duration or delta power (Table 2). However, the absence of statistically significant associations does not rule out potential confounding by age. Similarly, although sex was not significantly associated with the objective sleep parameters examined, the difference in sex distribution between the groups may represent a potential source of confounding.

Another consideration is the potential influence of hypnotic use. Although hypnotic use was significantly associated with WASO in the univariate correlation analysis, only three patients were receiving hypnotics (one GC user and two non-users), limiting the reliability and interpretation of this association. In a sensitivity analysis excluding these three patients, the differences in WASO, NREM stage N3 duration, and delta EEG power between GC users and non-users remained statistically significant (Supplementary Table S1). Nevertheless, given the potential effects of hypnotics on sleep architecture and the very small number of hypnotic users, residual confounding by hypnotic use cannot be excluded.

From a clinical perspective, these findings suggest that sleep disturbance in RA patients should not be attributed solely to disease activity, particularly in those receiving GC therapy. They also highlight the need to consider GC exposure as one of several potential factors associated with sleep disturbance in RA. However, whether reducing GC exposure improves sleep quality remains unclear and requires prospective evaluation. Persistent fatigue and impaired sleep remain major unmet needs in RA [18,38], and our findings suggest that GC exposure may contribute to these symptoms.

Several limitations of this exploratory pilot study should be considered. The small sample size, cross-sectional design, and single-center inpatient setting limit generalizability and preclude comprehensive multivariable adjustment. No formal post hoc power analysis was performed because of the exploratory nature of the study. Accordingly, neither independence from potential confounders nor a causal relationship between GC exposure and sleep alterations can be established. The single-center inpatient cohort may also have introduced selection bias, and the absence of a healthy control group limits the ability to determine whether the observed sleep characteristics are specific to GC exposure or reflect sleep abnormalities commonly observed in RA.

Although the daily GC dose at the time of assessment was available for all patients, detailed information regarding treatment duration and cumulative GC exposure could not be obtained because many patients had initiated GC therapy before referral to our institution. Furthermore, other important determinants of sleep in RA were not systematically assessed and therefore could not be adjusted for in the present analyses, including fatigue, anxiety, depression, nocturnal symptoms, and patient-reported disease impact. Although BMI and AHI were evaluated, obesity and obstructive sleep apnea syndrome were not exclusion criteria. Given the limited sample size, residual confounding by obesity and sleep-disordered breathing cannot be excluded.

Objective sleep assessment was performed using a single-channel EEG device rather than conventional polysomnography. Although the SLEEP SCOPE system has been validated against conventional polysomnography for clinical sleep staging [23,24,25,27,28], discrimination of N3 sleep remains less accurate than full polysomnography because sleep staging is based on a single EEG channel without electrooculography or electromyography. Therefore, some degree of sleep-stage misclassification cannot be excluded. In addition, sleep assessments were conducted in an inpatient setting, where unfamiliar surroundings, hospital routines, nighttime care, and ambient noise may have influenced sleep. Although recordings were performed at least two weeks after admission to allow acclimatization, hospitalization-related influences cannot be completely excluded.

Despite these limitations, the present findings provide preliminary evidence of an association between GC exposure and impaired sleep architecture in patients with RA. The consistency of the findings across several objective sleep parameters supports further investigation of this potentially underrecognized association in larger, appropriately adjusted longitudinal studies.

## 5. Conclusions

The present findings indicate that GC exposure is associated with impaired sleep continuity and reduced deep sleep in patients with RA, despite comparable disease activity between GC users and non-users. Given the exploratory cross-sectional design, these findings should be interpreted cautiously. Larger longitudinal studies are warranted to confirm these associations.

## Figures and Tables

**Table 1:** Comparison of clinical characteristics, sleep parameters, and autonomic function between glucocorticoid users and non-users.

Variable	GC Users	GC Non-Users	*p* Value
No.	9	11	-
Age, years	71.0 (64.3–76.5)	61.0 (52.8–66.8)	0.159
Female, no. (%)	6 (66.7)	10 (90.9)	0.285
BMI, kg/m^2^	21.9 (19.7–24.0)	26.0 (23.0–27.1)	0.053
RA duration, years	2.0 (1.0–7.3)	8.0 (1.0–12.5)	0.330
Diabetes mellitus, no. (%)	1 (11.1)	2 (18.2)	>0.999
Hypertension, no. (%)	5 (55.6)	5 (45.5)	>0.999
Hypnotic use, no. (%)	1 (11.1)	2 (18.2)	>0.999
eGFR, mL/min/1.73 m^2^	80.9 (56.4–86.6)	65.0 (54.3–89.3)	0.518
GC dose, mg/day	7.5 (4.3–10.0)	-	-
MTX use, no. (%)	4 (44.4)	7 (63.6)	0.653
MTX dose, mg/week	0.0 (0.0–8.0)	6.0 (0.0–7.5)	0.599
Biologic DMARD use, no. (%)	4 (44.4)	10 (90.9)	0.050
Biologic DMARD class, no.	TNF 1, IL-6 1, CTLA-4 2	TNF 7, IL-6 2, CTLA-4 1	-
VAS, mm	20.0 (10.0–50.0)	20.0 (11.3–37.5)	0.701
DAS28-CRP	2.9 (2.1–4.7)	3.0 (2.5–4.1)	0.970
AHI, events/hour	10.0 (1.8–21.8)	13.0 (3.3–20.0)	0.761
Sleep parameters			-
Total sleep time, min.	288.0 (257.5–414.8)	354.0 (277.5–413.8)	0.569
Sleep latency, min.	34.5 (20.4–40.5)	47.0 (22.3–54.9)	0.342
WASO, min.	145.0 (50.1–185.4)	34.0 (21.8–101.9)	0.011
Sleep efficiency, %	55.9 (52.3–81.5)	74.5 (63.7–83.7)	0.087
REM sleep, min.	70.0 (38.4–108.4)	74.0 (43.3–106.8)	0.970
NREM sleep, min.	227.5 (190.6–296.8)	285.5 (207.8–328.3)	0.342
NREM stage N1, min.	56.5 (48.6–141.4)	51.0 (45.3–68.1)	0.470
NREM stage N2, min.	136.0 (64.0–188.8)	159.0 (126.0–215.1)	0.305
NREM stage N3, min.	2.0 (0.0–3.9)	15.0 (6.0–39.4)	0.010
Delta EEG power during first sleep cycle, µV^2^	54,747.4 (26,068.2–70,629.6)	95,222.1 (66,543.9–200,068.3)	0.014
Autonomic function			
Nighttime-to-daytime LF/HF ratio	1.45 (0.92–1.62)	0.62 (0.50–1.08)	0.070

Data are presented as median (interquartile range) or number (%). GC, glucocorticoid; BMI, body mass index; RA, rheumatoid arthritis; eGFR, estimated glomerular filtration rate; MTX, methotrexate; DMARD, disease-modifying antirheumatic drug; VAS, visual analog scale; DAS, disease activity score; AHI, apnea–hypopnea index; CRP, C-reactive protein; EEG, electroencephalography; WASO, wake after sleep onset; NREM, non-rapid eye movement; REM, rapid eye movement; LF, low frequency; HF, high frequency.

**Table 2:** Spearman correlations between objective sleep parameters and clinical variables in patients with RA.

	WASO		NREM Stage N3 Duration		Delta EEG Power During First Sleep Cycle	
	ρ	*p*	ρ	*p*	ρ	*p*
Age	0.518	0.024	−0.146	0.524	−0.195	0.395
Sex (male/female = 0/1)	−0.119	0.603	0.198	0.389	0.173	0.450
BMI	−0.105	0.649	0.489	0.033	0.296	0.197
RA duration	−0.201	0.380	0.259	0.259	0.070	0.752
Diabetes (0/1)	−0.170	0.459	0.135	0.555	0.036	0.874
Hypertension (0/1)	0.252	0.273	0.185	0.421	−0.017	0.940
Hypnotic use (0/1)	−0.488	0.033	0.330	0.151	0.130	0.571
eGFR	0.051	0.736	−0.168	0.463	−0.060	0.793
GC use (0/1)	0.584	0.011	−0.592	0.010	−0.566	0.014
GC dose	0.642	0.005	−0.617	0.007	−0.647	0.005
MTX use (0/1)	−0.192	0.403	0.141	0.538	0.183	0.425
Biologic DMARD use (0/1)	−0.379	0.099	0.547	0.017	0.360	0.117
VAS	0.141	0.538	−0.097	0.671	−0.092	0.689
DAS28-CRP	0.073	0.751	−0.064	0.718	0.039	0.865
AHI	0.409	0.075	0.126	0.582	−0.301	0.190
Nighttime-to-daytime LF/HF ratio	0.450	0.063	−0.268	0.269	−0.112	0.643

WASO, wake after sleep onset; NREM, non-rapid eye movement; EEG, electroencephalography; BMI, body mass index; RA, rheumatoid arthritis; eGFR, estimated glomerular filtration rate; GC, glucocorticoid; MTX, methotrexate; DMARD, disease-modifying antirheumatic drug; VAS, visual analog scale; DAS28-CRP, Disease Activity Score in 28 joints based on C-reactive protein; AHI, apnea–hypopnea index; LF, low frequency; HF, high frequency.

## Data Availability

Data supporting the findings of this study are available upon request from the corresponding author (S.Y.).

## Supplementary Materials

The following supporting information can be downloaded at: https://www.mdpi.com/article/10.3390/jcm15166331/s1, Supplementary Table S1: Sensitivity analysis after excluding patients using hypnotics; Supplementary Table S2-1: Exploratory multivariable linear regression models for WASO; Supplementary Table S2-2: Exploratory multivariable linear regression models for NREM stage N3 duration; Supplementary Table S2-3: Exploratory multivariable linear regression models for delta EEG power during the first sleep cycle; Supplementary Table S3: Exploratory Spearman correlation analysis between GC dose and objective sleep parameters among GC users (n = 9).
