# Supplementary material for: Association Between Glucocorticoid Exposure and Objective Sleep Architecture in Rheumatoid Arthritis: An Exploratory Pilot EEG Study
Source: J Clin Med. 2026 Aug 16;15(16):6331. doi: 10.3390/jcm15166331 (PMC13513886; doi:10.3390/jcm15166331)
Supplement: Supplementary file 1 [file jcm-15-06331-s001.zip › jcm-4472827-supplementary.pdf]

Supplementary Table S1. Sensitivity analysis after excluding patients receiving hypnotics

Variable	GC users	GC non-users	*p* value
No.	8	9	-
WASO	161.3 (78.0–188.8)	38.5 (28.6–114.9)	0.021
NREM stage N3 duration, min.	1.3 (0.0–5.3)	15.5 (10.9–50.6)	0.006
Delta EEG power during first sleep cycle, μV^2	44,400.0 (25,776.1–72,209.9)	107,641.5 (63,231.7–157,615.6)	0.034

Data are presented as median (interquartile range). Abbreviations: GC, glucocorticoid; WASO, wake after sleep onset; NREM, non-rapid eye movement; EEG, electroencephalography

Supplementary Table S2-1. Exploratory multivariable linear regression models for WASO

Variable	Model 1		Model 2		Model 3		Model 4	
	β	*p*	β	*p*	β	*p*	β	*p*
GC use (0/1)	0.421	0.051	0.538	0.026	0.426	0.087	0.550	0.007
Age	0.340	0.109						
RA duration			0.033	0.883				
Biologic DMARD use (0/1)					0.198	0.409		
AHI							0.426	0.029
Model *p* value (R^2)	0.017 (0.381)		0.063 (0.277)		0.045 (0.306)		0.006 (0.457)	

Model 1: adjusted for age. Model 2: adjusted for RA duration. Model 3: adjusted for biologic DMARD use. Model 4: adjusted for AHI. Values are standardized regression coefficients (β) obtained from exploratory multivariable linear regression analyses. R^2, coefficient of determination. WASO, wake after sleep onset; GC, glucocorticoid; RA, rheumatoid arthritis; DMARD, disease-modifying antirheumatic drug; AHI, apnea-hypopnea index.

Supplementary Table S2-2. Exploratory multivariable linear regression models for NREM stage N3 duration

Variable	Model 1		Model 2		Model 3		Model 4	
	β	*p*	β	*p*	β	*p*	β	*p*
GC use (0/1)	−0.546	0.020	−0.499	0.032	−0.376	0.104	−0.550	0.015
Age	−0.018	0.932						
RA duration			0.146	0.503				
Biologic DMARD use (0/1)					−0.348	0.129		
AHI							0.021	0.917
Model *p* value (R^2)	0.046 (0.305)		0.036 (0.323)		0.014 (0.395)		0.046 (0.305)	

Model 1: adjusted for age. Model 2: adjusted for RA duration. Model 3: adjusted for biologic DMARD use. Model 4: adjusted for AHI. Values are standardized regression coefficients (β) obtained from exploratory multivariable linear regression analyses. R^2, coefficient of determination. NREM, non-rapid eye movement; GC, glucocorticoid; RA, rheumatoid arthritis; DMARD, disease-modifying antirheumatic drug; AHI, apnea-hypopnea index.

Supplementary Table S2-3. Exploratory multivariable linear regression models for delta EEG power during the first sleep cycle

Variable	Model 1		Model 2		Model 3		Model 4	
	β	*p*	β	*p*	β	*p*	β	*p*
GC use (0/1)	−0.618	0.007	−0.696	0.003	−0.554	0.021	−0.641	0.001
Age	−0.010	0.961						
RA duration			−0.209	0.304				
Biologic DMARD use (0/1)					−0.134	0.546		
AHI							−0.361	0.048
Model *p* value (R^2)	0.016 (0.386)		0.009 (0.424)		0.013 (0.399)		0.002 (0.516)	

Model 1: adjusted for age. Model 2: adjusted for RA duration. Model 3: adjusted for biologic DMARD use. Model 4: adjusted for AHI. Values are standardized regression coefficients (β) obtained from exploratory multivariable linear regression analyses. R^2, coefficient of determination. EEG, electroencephalography; GC, glucocorticoid; RA, rheumatoid arthritis; DMARD, disease-modifying antirheumatic drug; AHI, apnea-hypopnea index.

Supplementary Table S3. Exploratory Spearman correlation analysis between GC dose and objective sleep parameters among GC users (n = 9)

	ϱ	*p*
WASO	0.579	0.102
NREM stage N3 duration	−0.458	0.195
Delta EEG power during the first sleep cycle	−0.712	0.044

GC, glucocorticoid; WASO, wake after sleep onset; NREM, non-rapid eye movement; EEG, electroencephalography.
